# Kala-azar in Darfur: Evidence for indigenous transmission in Al-Malha Locality, North Darfur, western Sudan

**DOI:** 10.1186/s13071-018-2746-3

**Published:** 2018-03-06

**Authors:** Mohammed A. Mohammed, Noteila M. Khalid, Mariam A. Aboud

**Affiliations:** 1 Department of Biological Sciences, Al-Fashir University, Al-Fashir, Sudan; 2 Department of Zoology, Ibn Sina University, Khartoum, Sudan; 3 Department of Biology and Biological Technology, Faculty of Science and Technology, Al-Neelain University, Khartoum, Sudan

**Keywords:** Kala-azar, Sand fly vectors, North Darfur, Sudan

## Abstract

**Background:**

Recent reports showed high numbers of visceral leishmaniasis cases in North Darfur, western Sudan. Due to a lack of previous studies, no information is available on local transmission of the disease in these areas. Therefore, a pilot entomological and epidemiological study was conducted in Al-Malha Locality during the year 2013, to investigate possibility of local transmission and places and times of the year where and when people contract the infection.

**Methods:**

Kala-azar incidence data were obtained from records of Ministry of Health, North Darfur; Al-Malha rural hospital; and the Federal Ministry of Health, Division of Communicable and Non-communicable Diseases. Sand flies were collected using sticky paper and rodent burrow traps from five different microhabitats during three different phases of the year. Species identification was undertaken using appropriate taxonomic keys. Data were statistically analyzed to determine the distribution of kala-azar among different age groups and between sexes, and to compare the species richness and distribution of different sandfly species between the different microhabitats.

**Results:**

The most affected age groups with kala-azar during the period 2013–2016 were children between one and five years old and those under one year. Females were found to be more affected than males. A total of 918 sand fly specimens were collected using sticky paper and rodent burrow traps from five microhabitats. Identified specimens belong to 13 species; 5 *Phlebotomus* and 8 *Sergentomyia*. *Phlebotomus orientalis*, the principal vector of visceral leishmaniasis (VL) in Sudan and other East African countries, was found for the first time in the area. No other known vector of VL was found in the collection. The highest number of sand flies was recorded during the summer season (63%), with *S. antennata* (48%) and *S. schwetzi* (24.1%) being the most abundant species. Among *Phlebotomus* species, *P. orientalis* showed relatively high density (8.6%). A dry seasonal water course (called “Khor”) seems to be the most preferred habitat for most of the sand fly species since most of the collections (41.2%) were made from this site, followed by the rodent burrows.

**Conclusions:**

The presence of *P. orientalis* and the high prevalence of VL in infants in the Al-Malha area provide the first evidence for local transmission of the parasite causing kala-azar in Darfur. Transmission is probably occurring during summer near the woodland where a high density of the vector was recorded. As a pre-requisite for designing effective control of VL in North Darfur, large scale entomological and epidemiological studies are recommended.

## Background

Leishmaniasis is an important vector-borne disease, endemic in 98 countries, 72 of which are in the third world [1]. About 90% of the estimated annual worldwide cases of visceral leishmaniasis (VL) occur in Brazil, Bangladesh, India, Nepal, Sudan, Southern Sudan, Uganda and Ethiopia [2].

Most leishmaniasis cases are zoonotic, transmitted to humans from animal reservoir hosts by the bite of an infected female sand fly vector [3]. However, in East Africa including Sudan, anthroponotic transmission of the parasite causing VL is thought to occur, especially in villages with a significant presence of *A. seyal* and/or *B. aegyptiaca* trees or during epidemic situations [4, 5]. Among domestic animals, dogs have been found infected by the parasite, but its role in the epidemiology of VL remains to be determined [6].

In Sudan, leishmaniasis represents a serious health problem. Cutaneous leishmaniasis occurs in a fluctuating pattern in the country mainly in the west, central and northern parts [7]. Visceral leishmaniasis or kala-azar is endemic in savannah areas extending from the Sudanese-Ethiopian border in the east to the banks of the White Nile in the west, and from Kassala in the northeast towards the Blue Nile State in the south, with scattered foci in the Nuba Mountains and Darfur [8]. Recently, the disease has revived in a dormant focus in the White Nile State, Central Sudan which may represent an extension of the disease’s geographical range [9].

Darfur is a large region in western Sudan, extending over 190 square miles (about the size of Spain). It is bordered to the northwest by Libya, to the west by Chad and to the southwest by Central African Republic. The region is subdivided into three main federal states (North, West and South Darfur). Due to climate change and political reasons, the region has recently suffered enormously from severe civil war and armed conflicts that resulted in the displacement of millions of people [10].

Darfur is well known to be endemic for cutaneous leishmaniasis [6]. The state was considered to be free of visceral leishmaniasis. However, in the past few years, records of the Ministry of Health (FMoH) in Sudan indicated an alarming incidence of VL in Darfur with about 662 reported cases in the period 2006–2010 to 886 cases in 2011–2016 in North Darfur State. Unfortunately, no publications are available on the disease incidence. Furthermore, it is unknown whether transmission occurs locally or if infection is acquired in other regions of the country, e.g. Gedarif State where Darfurian people go for agriculture.

The current study was conducted in Al-Malha Locality (a VL endemic site) during 2013 to examine the possibility of local transmission of VL through investigation of the sand fly fauna, presence of the sand fly vectors, abundance and seasonal distribution. Additionally, work was done to determine incidence rate and age/sex distribution of VL among the different age groups.

## Methods

### Study area

**Fig. 1:**
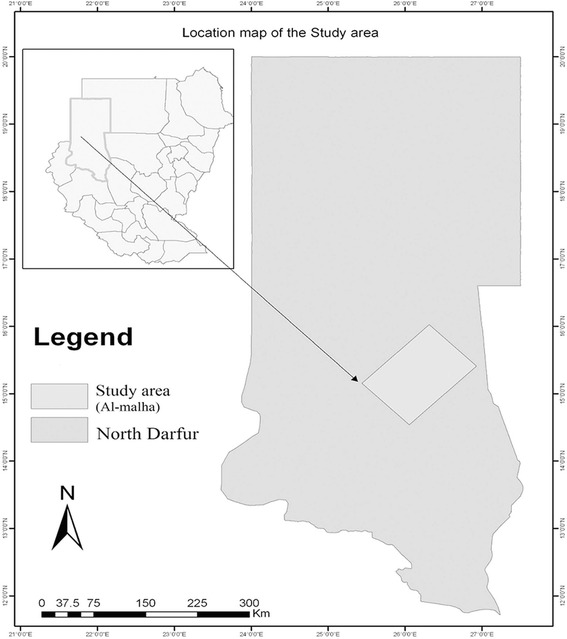
Map of Darfur showing the location of Al-Malha area. The inset map shows the location of Darfur State in Sudan

**Fig. 2:**
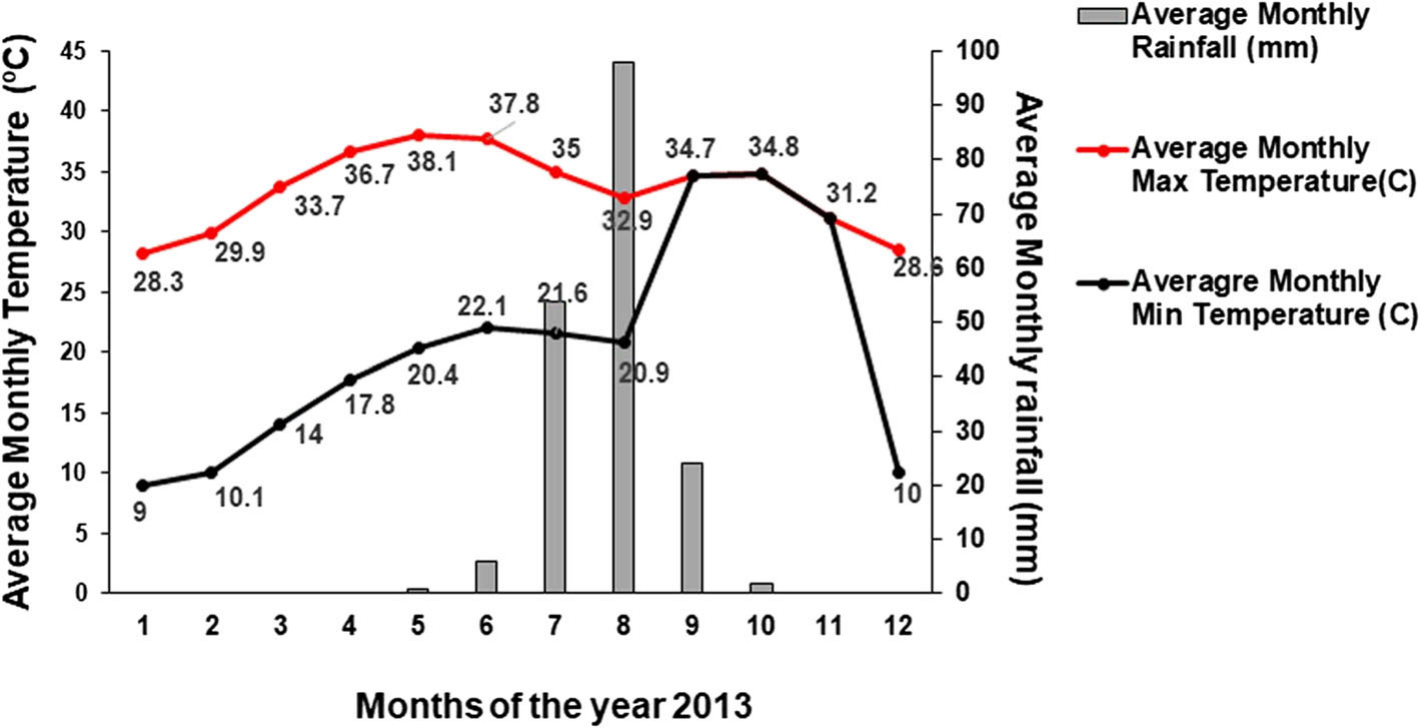
Monthly fluctuation of average rainfall (mm) and maximum and minimum temperature at Al-Malha Locality, North Darfur, western Sudan, during 2013

Al-Malha (15°13'40.74"N, 26°15'00.99"E) is a small rural locality, that lies about 215 km north of Al-Fasher town, the capital of North Darfur (Fig. 1). Extending over an area of about 22,000 km^2^, Al-Malha Locality consists of small villages scattered near water and pasture resources, around volcanic mountains. The area is characterized by a long dry season (October-June), followed by a short rainy season (July-September). During the rainy season water drains down seasonal streams known as “Khors” which run through rocky valleys and shallow soil. A cold winter starts around October and ends in December. In March, the area becomes warm and quite dusty. The annual mean maximum temperature varies from 28.3 to 38.1°C, while the annual mean minimum temperature varies from 9.0 to 22.1 °C. The average annual rainfall received by the area during 2013 was 30 mm (https://www.worldweatheronline.com) (Fig. 2).

### Kala-azar data collection

Kala-azar incidence data were collected from the records of Al-Malha Rural Hospital, the Ministry of Health, North Darfur and the Federal Ministry of Health, Division of Communicable and Non-communicable Diseases. Only serologically (rK39 RDT) and clinically confirmed VL cases were included in the analysis.

### Sandfly collection sites

**Fig. 3:**
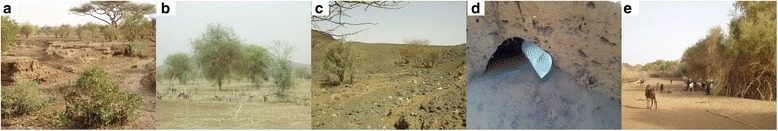
Sites used for sampling sand flies at Al-Malha Locality, North Darfur, western Sudan, during 2013. **a** “Khor”. **b** Woodland. **c** Hill. **d** Rodent burrow. **e** Village

The following five sites representing different microhabitats in the area were designated for routine sampling of sand flies (Fig. 3a-e):

- “Khor”: This is the main water course that runs through different parts of the study area. It usually floods during the rainy season and then dries up during summer. Its bottom is characterized by cracked clay soil, rich in rodent and other animal burrows.
- Woodland: Found in the centre of the plain covering about 20 km^2^, this site is characterized by clay soil and scattered trees and shrubs which are dominated by *Dosscis sengalensis*, *Capparis delielua*, *Acacia nubica*, *Acacia tortilis* and *Balanities agyptiaca*. Some villagers live about 300–2000 m from the edge of the woodland, while others live inside this site, especially during dry summer season.
- The Hill: An elevated (860.45 m), completely eroded area around the basin of the Wadi which is a seasonal water-course. Covered with rocks and limestone soil at top of the slope, and mixed sandy soil at the bottom of the slope where scattered trees like *Acacia nubia*, *Acacia tortilis* and some grasses are found.
- Rodent burrows: Small holes made by rodents for resting and breading, mainly found near small bushes and shrubs in the woodland and Khor sites.
- A village (called Zakaria village) composed of scattered huts made of wood and grass, surrounded by woody fences found scattered in an elevated area (851.61 m) near the basin of the Wadi.

### Sand fly collection and preservation

Sand flies were collected twice per month using Pyrethrum Spray catches (PSC), sticky paper and rodent burrow traps as described by Alexander [11]. At each time point of the study, 5 huts were surveyed by PSC and 10 sticky paper traps were used per site. The traps were fixed overnight from 6:00 pm to 6:00 am. Each trap is made of a white A4 paper sheet, soaked with castor oil on both sides and fixed on sticks held vertically at a height of 20 cm above the ground level. For collections made from rodent burrows, oil soaked papers were rolled and inserted into the burrow.

Captured sand flies were removed from the paper traps using fine brushes, washed with distilled water and detergent and preserved in 70% ethanol for identification. Before identification, the head and the abdominal segments of each sand fly specimen were dissected and glass-mounted using locally made Berleze’s medium. The specimens were then identified to the species level using keys proposed by Kirk & Lewis [12], Quate [13] and Abonnenc & Minter [14].

### Statistical analysis

Data were analyzed using SPSS statistical package (v. 20). Data was not normally distributed. Therefore, the nonparametric Mann-Whitney test was used to compare the median number of VL cases among male and female patients, while the Chi-square test was used to test the distribution of cases among the different age groups.

The Shannon-Weiner (H), evenness (E) and species richness (S) diversity indices of the collected sand flies within different microhabitats were estimated according to Heip et al. [15] using Microsoft Excel sheets (MS Office 2015) as follows:

$$ H=-\sum \limits_{i=1}^s\  Pi\ln pi $$

where, Shannon-Weiner index (H) is a quantitative estimate of biological variability as described by Heip et al. [15]. It reflects the number of different species and how evenly the individuals are distributed among these species. The value of the index increases as diversity increases. The relative abundance of each species (*p*_*i*_) is the proportion of species *i* relative to the total number of species.

Species evenness (*E*) **= ***H*/ln(*S*) indicates how evenly the individuals in the community are distributed among species. The index ranges between 0 and 1; lower values indicate the presence of dominant species.

Species richness (*S*) is the total number of different species in the study area.

Species composition was compared between the different collection sites using the Jaccard index of similarity (*SC*_*j*_) which is calculated using the formula:

$$ {SC}_j=\left(\mathrm{c}/\mathrm{A}+\mathrm{B}-\mathrm{c}\right)\times 100 $$

where, A and B are the richness values of two different collection sites and c is the number of species found in both sites; higher richness indicates higher biodiversity.

## Results

### Incidence of kala-azar in Al-Malha Locality

**Fig. 4:**
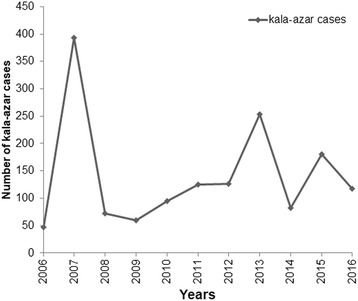
Numbers of confirmed cases of kala-azar reported in North Darfur, western Sudan, during the period 2006–2016. Data were obtained from Division of Communicable and Non-communicable Diseases, Federal Ministry of Health

Figure 4 shows the fluctuation of the number of kala-azar cases in North Darfur State during the period 2006–2016 according to reported cases from the Federal Ministry of Health, Division of Communicable and Non-communicable Diseases. Only detailed data from records of Al-Malha Rural Hospital were analyzed in this study.

**Fig. 5:**
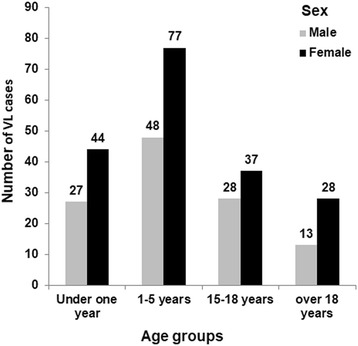
Age/sex distribution of kala-azar cases among all cases reported to Al-Malha Rural Hospital during the period 2013–2016

The number of male and female VL cases reported by the locality rural hospital during the period 2013–2016 were analyzed (Fig. 5). The most affected age groups were children between 1 and 5 years old (125 cases) and those under 1year (71 cases) (*χ*^2^ = 49.94, *df*= 3 and *P* < 0.05).

Females were more affected by VL than males, with a total number of infected females of 186 and 116 males during the period 2013–2016 which was significantly higher than that of males (Mann-Whitney U-test: *U* = 6374.50, *Z* = -6.019, *P* = *P* < 0.0001)

### Sand fly species composition and relative abundance in Al-Malha Locality

Nine hundred and eighteen sand flies were collected and identified during this study period. The collected samples belong to 13 sand fly species, 5 of which were *Phlebotomus* (*P*. *orientalis*, *P*. *papatasi*, *P*. *bergeroti*, *P*. *duboscqi* and *P. heischi*) and 8 were *Sergentomyia* (*S*. *antennata*, *S*. *schwetzi*, *S*. *clydei*, *S*. *bedfordi*, *S*. *adleri*, *S*. *affinis*, *S. squamipleuris* and *S. africana*). A high abundance rate of *Sergentomyia* species was recorded (89.5%) with males (53%) outnumbering females (47%). However, the male/female ratio differed among species: *P. orientalis*: 5.1; *P*. *papatasi*: 1.7; *S*. *antennata*: 0.94; *S*. *schwetzi*: 0.55; and *S*. *clydai*: 1.9.

*Sergentomyia antennata* was the predominant species represented by (47.9%) of the total collections, followed by *S*. *schwetzi* (19.9%) and *S*. *clydei* (14.5%). Among *Phlebotomus* species, *P*. *orientalis* was the most abundant species (8%). Table 1 summarizes the sex ratio, total number, relative abundance and total number of flies per m^2^ for each of the collected sand fly species.

**Table 1:** Species composition, sex ratio, relative abundance and total number of sand flies per m^2^ collected from Al-Malha Locality, North Darfur, Sudan during the year 2013

Species	No. of male flies	No. of female flies	Relative abundance (%)^a^	Total no. of flies/m^2 b^
*P*. (*Larroussius*) *orientalis*	61	12	8	1.39
*P*. (*Phlebotomus*) *duboscqi*	2	0	0.22	0.038
*P*. (*Phlebotomus*) *bergeroti*	3	0	0.33	0.057
*P*. (*Phlebotomus*) *papatasi*	10	6	1.74	0.305
*P.* (*Phlebotomus) heischi*	2	0	0.22	0.038
*S*. (*Sergentomyia*) *antennata*	213	227	47.9	8.38
*S*. (*Sergentomyia*) *schwetzi*	65	118	19.9	3.486
*S*. (*Sergentomyia*) *bedfordi*	6	0	0.65	0.114
*S*. (*Sintonius*) *clydei*	87	46	14.5	2.533
*S*. (*Sintonius*) *adleri*	5	7	1.31	0.229
*S*. (*Sintonius*) *affinis*	8	0	0.87	0.152
*S*. (*Grassomyia*) *squamipleuris*	11	9	2.2	0.380
*S. *(*Parrotomyia) africana*	19	1	2.2	0.380
	487 (53%)	431 (47%)	100	17.49

^a^Relative abundance was calculated as the abundance of each species (total number), divided by the total abundance of all species combined

^b^Number of flies per m^2^ = number of collected specimens for each species per each trap (oil soaked A4 paper) × the number of A4 papers in 1m^2^ area

### Seasonal fluctuations of sand fly species from January to October 2013

The majority of sand flies (63.2 %) were collected during the summer season (March-June), with a higher abundance rate of *Sergentomyia* species (88.1%) than *Phlebotomus* species (11.9 %). Eighteen percent of collections were recorded in early dry season (January-February) and another 18% during the winter season (October-December). No collections were made during the rainy season (July-September) due to the inaccessibility of the collection sites which became completely covered by flood from different Khors (Table 2).

**Table 2:** Sand fly species composition and density (total number of collected flies per season) collected at three different phases of the year 2013 from Al-Malha Locality, North Darfur, Sudan

Species	Late winter (January-February)	Summer (March-June)	Early winter (October-December)	Total
*P*. (*Larroussius*) *orientalis*	19	50	4	73
*P*. (*Phlebotomus*) *duboscqi*	0	2	0	2
*P*. (*Phlebotomus*) *bergeroti*	0	3	0	3
*P*. (*Phlebotomus*) *papatasi*	1	14	1	16
*P.* (*Phlebotomus) heischi*	1	0	1	2
*S*. (*Sergentomyia*) *antennata*	85	279	76	440
*S*. (*Sergentomyia*) *schwetzi*	34	140	9	183
*S*. (*Sergentomyia*) *bedfordi*	1	5	0	6
*S*. (*Sintonius*) *clydei*	16	47	70	133
*S*. (*Sintonius*) *adleri*	2	6	4	12
*S*. (*Sintonius*) *affinis*	0	8	0	8
*S*. (*Grassomyia*) *squamipleuris*	9	10	1	20
*S. *(*Parrotomyia) africana*	2	16	2	20
	170 (18.5%)	580 (63.2%)	168 (18.3%)	918

**Fig. 6:**
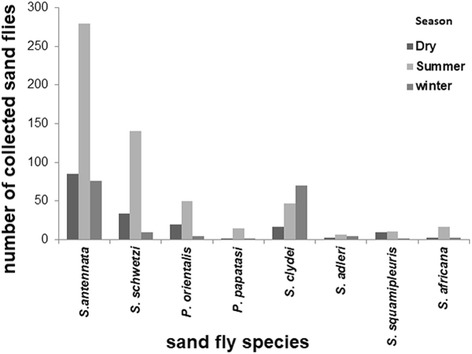
Seasonality of sand flies at Al-Malha Locality, North Darfur, western Sudan, during 2013

The most abundant species during summer were *S. antennata* (48%), *S. schwetzi* (24.1%), *P*. *orientalis* (8.6%) and *S. clydei* (8.1%). Most species were absent during winter; however, *S. antennata* (45.2%) and *S. clydei* (41.7%) were the most abundant during this season (Fig. 6).

*Phlebotomus orientalis* appeared in early dry season in the woodland and Khor habitats and reached its peak abundance in late summer. Significant fluctuations of the abundance of *P. orientalis* were observed in Khor, woodland, and rodent burrows.

**Fig. 7:**
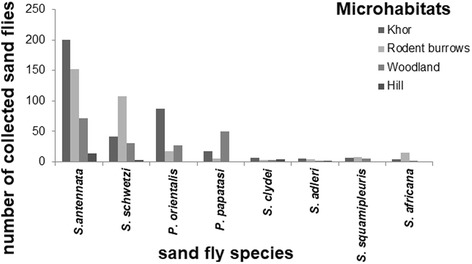
Comparison of numbers of sand flies captured at different microhabitats (Khor, woodland, hill, rodent burrows) of Al-Malha Locality, North Darfur, western Sudan, during 2013

*Phlebotomus papatasi* appeared at different habitats in small numbers in the summer season, while extremely small numbers of other *Phlebotomus* species (*P. duboscqi* and *P. bergeroti*) were rarely encountered in the collection (Fig. 7).

### Sand fly prevalence in the different collection microhabitats

A clear variation in the distribution of the collected sand fly species across the different microhabitats was observed, with 41.2% of the total collections being made from the Khor, followed by the rodent burrows (35.1%) and the woodland (20.9%) (Table 3). Although the woodland microhabitat showed less collection percentages and species richness (9 species) than the Khor and rodent burrows, this site resulted in the highest diversity indices (Table 4). This may be due to the dominance of *S*. *antennata* in the Khor (52.9%) and rodent burrows (47.2%). Other species showed different relative abundance at the different sites. For example, *S*. *schwetzi* was most abundant in rodent burrows (35.5%), *S*. *clydei* was most abundant in Khor (23%), while *P*. *orientalis* was most abundant in the woodland (26%) than in other sites. It seems that the eroded hill habitat was not preferred by most species since only 4 species were collected from this site (2.4%). The village showed the lowest density (0.44%) with only 4 specimens (2 *S*. *antennata* and 2 *S*. *clydei*) collected from this site using knockdown method (Table 3).

**Table 3:** Sand fly species composition and density collected from four different microhabitats during 2013 from Al-Malha Locality, North Darfur, Sudan

Species	Khor	Rodent burrows	Woodland	Hill	Village	Total
*P*. (*Larroussius*) *orientalis*	18	5	50	0	0	73
*P*. (*Phlebotomus*) *duboscqi*	1	1	0	0	0	2
*P*. (*Phlebotomus*) *bergeroti*	0	3	0	0	0	3
*P*. (*Phlebotomus*) *papatasi*	6	3	3	4	0	16
*P*. (*Phlebotomus) heischi*	1	0	1	0	0	2
*S*. (*Sergentomyia*) *antennata*	200	152	72	14	2	440
*S*. (*Sergentomyia*) *schwetzi*	41	108	31	3	0	183
*S*. (*Sergentomyia*) *bedfordi*	3	3	0	0	0	6
*S*. (*Sintonius*) *clydei*	87	17	27	0	2	133
*S*. (*Sintonius*) *adleri*	5	4	2	1	0	12
*S*. (*Sintonius*) *affinis*	5	3	0	0	0	8
*S*. (*Grassomyia*) *squamipleuris*	7	8	5	0	0	20
*S. *(*Parrotomyia) africana*	4	15	1	0	0	20
	378 (41.2%)	322 (35.1%)	192 (20.9 %)	22 (2.4%)	4 (0.4%)	918

**Table 4:** Diversity indices for sand fly species collected from different microhabitats in Al-Malha Locality, North Darfur, during 2013

Diversity index	Khor	Rodent burrows	Woodland	Hill
Species evenness (E)	0.58	0.57	0.71	0.73
Species richness (S)	12	12	9	4
Shannon-Weiner diversity index (H)	1.43	1.42	1.55	1.01

The Jaccard index of similarity (*SC*_*j*_) showed a high species similarity between the Khor and rodent burrows (84.6%) and between the Khor and woodland (75%).

## Discussion

Studies on sand fly fauna and their seasonal distribution are crucial for predicting transmission of leishmaniasis, and for the successful implementation of control programs [16]. This study provides baseline data on sand fly ecology, distribution, and their expected role as vectors of leishmaniasis in Al-Malha Locality, North Darfur, West Sudan.

The analyzed numbers of kala-azar cases in Al-Malha Locality during 2013–2016 indicates a slightly higher incidence of disease among females than males in most age groups. The data also provided strong evidence for the indigenous nature of the kala-azar transmission in Al-Malha Locality since the most affected age groups were found to be children between one and five years, and those under one year. Studies on other parts of western Sudan reported a similar finding [17]. *Phlebotomus orientalis*, the vector of VL is known to be more abundant in *Acacia-Balanites* woodland and rarely associated with human dwellings [13, 18]. It seems that the presence of the village at the edge of the woodland makes the villagers more vulnerable to peridomestic transmission of the disease especially during the hot seasons of the year when most people sleep outdoors [19].

More information is needed about the source of infection, parasite strain and the expected reservoir hosts. According to the kala-azar risk map based on predicted vector distribution [20], western Sudan includes some of the high risk foci of VL. The current results confirm this predicition and provide the first evidence for active transmission of VL in the area.

In eastern Sudan, several studies provided evidence for the existence of zoonotic cycle of *L*. *donovani* in the woodlands of Dinder National Park [21, 22], with the possibility of the Egyptian mongoose (*Hyrpestes ichneumon*) acting as a sylvatic reservoir host [23]. Transmission has also been demonstrated to take place in the village habitat, although it is more likely that in these habitats transmission is probably anthroponotic [24].

Although western Sudan has been endemic for cutaneous leishmaniasis for a long time, no records are available on the epidemiology of the disease or its transmission cycle in the region. It is noteworthy that *P. papatasi*, the main vector of *L. major* in Sudan and North Africa, and *P. duboscqi* the main vector in West Africa, were collected in this study. The study area is rich with burrows of rodents and other small mammals that may be suitable reservoir hosts of *Leishmania* parasites. Therefore, detailed studies on the role of these vectors in the transmission and maintenance of CL in Darfur are recommended.

Thirteen sand fly species were recorded in this study, indicating the fauna richness of the area which can be attributed to the diversity of microhabitats and vegetation in the collection sites. Moreover, availability of rodents and other small animal burrows may provide suitable breeding and resting sites for many sand fly species.

In Sudan and other parts of the old world, most sand fly species including *P. orientalis*, show seasonal abundance with an increasing abundance during the dry season (January-May), reaching the peak in the early rainy season (April) and drastically drop in numbers during the wet season (August-November) [13, 18, 25]. Unfortunately, the authors were unable to access the study area during the rainy season and made collections so as to confirm such observations on seasonality of sand fly species in this far part of Sudan.

The abundance of *P*. *orientalis* in the woodland was also reported by Quate [13] and Elnaiem & Osman [26]. They confirmed that *P*. *orientalis* thrives in habitats dominated by *Acacia seyal/Balanites aegyptiaca*. In a subsequent publication, the same research group showed a high abundance of VL and active transmission of VL in the village and presented evidence for inter-annual fluctuation of vector density [5]. No sand flies were collected from indoor knockdown collections. For *P*. *orientalis*, this result agrees with previous reports which indicated that the vector rarely rests at indoor sites [18]. However, for other species the results are surprising since *Sergentomyia* spp. and *P*. *papatasi* are known to prefer indoor resting sites [27]. The preference of *S. schwetzi* in the rodent burrows may indicate a close association between this sand fly species and rodents as a preferred feeding host. Moreover, the relatively more humid microclimatic conditions within these burrows present a more suitable ecological niche for this sand fly species. This has also been observed long time ago by Kirk & Lewis [28].

The predominance of *S. antennata* throughout the year is in accordance to the findings of Quate [13] in Polich area who mentioned that *S. antennata* is a non-seasonal species.

The overall sex ratio of most of the collected sand fly species in this study showed that males were more abundant than females, especially for *P. orientalis*. This finding corroborates the findings of Doha & Samy [29] who also observed a high male/female sex ratio for *P*. *orientalis* in Al-Baha, Saudi Arabia. This biased sex ratio is probably due to difference in behavior of either sexes or bias introduced by collection methods [30]. It may also be that females are not attracted to non-baited oil sticky traps [31].

## Conclusions

Results obtained in this study are essential to understand the abundance, and seasonality of sand fly species in Al-Malha Locality which represent an essential pre-requisite of the control of leishmaniasis through the control of its sand fly vectors. However, many factors interfere with the fulfillment of the objectives of the study. This includes, for example, the refusal of some Al-Malha residents on the usage of light traps thinking that it may attract the fighting groups. Also, data on seasonality of sand fly species was missing due the inaccessibility of the collection sites during the flood season, in addition to difficulties in obtaining detailed records of kala-azar incidences which add some limitations to interpretation of transmission data. Large-scale entomological surveillance in North Darfur and surrounding areas is highly needed to elucidate the spatial and temporal distribution of sand fly vectors and design effective control policy.

## Abbreviations

MoH: Ministry of Health
PSC: Pyrethrum spray catches
VL: Visceral leishmaniasis

## References

[1] Alvar J, Vélez ID, Bern C, Herrero M, Desjeux P, Cano J (2012). Leishmaniasis worldwide and global estimates of its incidence. PLoS One.

[2] Chappuis F, Sundar S, Hailu A, Ghalib H, Rijal S, Peeling RW, et al. Visceral leishmaniasis: what are the needs for diagnosis, treatment and control? Nat Rev Microbiol. 2007;5:873–82. 10.1038/nrmicro1748 17938629

[3] Ashford RW (2000). The leishmaniases as emerging and reemerging zoonoses. Int J Parasitol.

[4] Dereure J, El-Safi SH, Bucheton B, Boni M, Kheir MM, Davoust B, et al. Visceral leishmaniasis in eastern Sudan: parasite identification in humans and dogs; host-parasite relationships. Microbes Infect. 2003;5:1103–8. 10.1016/j.micinf.2003.07.003 14554251

[5] Hassan MM, Elraba'a FMA, Ward RD, Maingon RDC, Elnaiem DA (2004). Detection of high rates of in-village transmission of *Leishmania donovani* in eastern Sudan. Acta Trop.

[6] Desjeux P (2001). The increase in risk factors for leishmaniasis worldwide. Trans R Soc Trop Med Hyg.

[7] El-Hassan AM, Zijlstra EE. Leishmaniasis in Sudan. Cutaneous leishmaniasis. Trans R Soc Trop Med Hyg. 2001;95(Suppl. 1):S1–17. 10.1016/s0035-9203(01)90216-0 11370248

[8] El-Hassan AM, Zijlstra EE (2001). Leishmaniasis in Sudan. Visceral leishmaniasis. Trans R Soc Trop Med Hyg.

[9] Khalil EAG, Musa AM, Elgawi SHH, Meshasha A (2008). Revival of a focus of visceral leishmaniasis in central Sudan. Ann Trop Med Parasitol.

[10] O’Fahey RS (2004). Conflict in Darfur: Historical and contemporary perspectices. In: Environmental Degradation as a Cause of Conflict in Darfur.

[11] Alexander B (2000). Sampling methods for phlebotomine sand flies. Med Vet Entomol.

[12] Kirk RO, Lewis DJ (1951). The Phlebotominae of the Ethiopian Region. Trans R Soc Trop Med Hyg.

[13] Quate LW (1964). Leishmaniasis in the Sudan Republic 19. *Phlebotomus* sand flies of the Paloich area in the Sudan (Diptera: Psychodidae). J Med Entomol.

[14] Abonnenc E, Minter DM (1965). Bilingual key for the identification of sandfly of the Eithiopian region (French and English). ORSTON, Sreie Entomol Med.

[15] Heip CHR, Herman PMJ, Soetaert K (1998). Indices of diversity and evenness. Oceanis.

[16] Cross ER, Hyams KC (1996). The potential effect of global warming on the geographic and seasonal distribution of *Phlebotomus papatasi* in Southwest Asia. Environ Health Perspect.

[17] Osman AA (2011). Epidemiology of leishmaniasis in south Kordofan region, western Sudan. Res J Med Scien.

[18] Elnaiem DA, Hassan HK, Ward RD (1997). Phlebotomine sand flies in a focus of visceral leishmaniasis in a border area of eastern Sudan. Ann Trop Med Parasitol.

[19] Oryan A, Akbari M (2016). Worldwide risk factors in leishmaniasis. Asian Pac J Trop Med.

[20] Thomson MC, Elnaiem DA, Ashford RW, Connor SJ. Towards a kala-azar risk map for Sudan: mapping the potential distribution of *Phlebotomus orientalis* using digital data of environmental variables. Trop Med Int Health. 1999;4:105–13. 10.1046/j.1365-3156.1999.00368.x 10206264

[21] Elnaiem DA, Ward RD, Hassan HK, Miles MA, Frame IA (1998). Infection rates of *Leishmania donovani* in *Phlebotomus orientalis* from a focus of visceral leishmaniasis in eastern Sudan. Ann Trop Med Parasitol.

[22] Elnaiem DE, Hassan HK, Maingon RD, Killick-Kendrick R, Ward RD, Osman OF (2011). A possible role for *Phlebotomus* (*Anaphlebotomus*) *rodhaini* (Parrot, 1930) in transmission of *Leishmania donovani*. Parasit Vectors.

[23] Elnaiem DA, Hassan MM, Maingon R, Nureldin GH, Mekawi AM, Miles M, Ward RD (2001). The Egyptian mongoose, *Herpestes ichneumon*, is a possible reservoir host of visceral leishmaniasis in eastern Sudan. Parasitology..

[24] Elnaiem DA (2011). Ecology and control of the sand fly vectors of *Leishmania donovani* in East Africa, with special emphasis on *Phlebotomus orientalis*. J Vector Ecol.

[25] Yared S, Gebresilassie A, Akililu E, Balkew M, Warburg A, Hailu A, Gebre-Michael T (2017). Habitat preference and seasonal dynamics of *Phlebotomus orientalis* in urban and semi-urban areas of kala-azar endemic district of Kafta Humera, northwest Ethiopia. Acta Trop.

[26] Elnaiem DA, Osman OF (1998). Evidence for active transmission of visceral leishmaniasis within a village in eastern Sudan. Acta Trop.

[27] Ebrahimi S, Bordbar A, Rastaghi ARE, Parvizi P (2016). Spatial distribution of sand fly species (Psychodidae: Phlebtominae), ecological niche, and climatic regionalization in zoonotic foci of cutaneous leishmaniasis, southwest of Iran. J Vector Ecol.

[28] Kirk R, Lewis DJ. Studies in leishmaniasis in the Anglo-Egyptian Sudan. III. The sand flies (*Phlebotomus*) of the Sudan. Trans R Soc Trop Med Hyg. 1940;33:623–34.

[29] Doha SA, Samy AM (2010). Bionomics of phlebotomine sand flies (Diptera: Psychodidae) in the Province of Al-Baha, Saudi Arabia. Mem Inst Oswaldo Cruz.

[30] Lane RP, Pile MM, Amerasinghe FP (1990). Anthropophagy and aggregation behaviour of the sand fly *Phlebotomus argentipes* in Sri Lanka. Med Vet Entomol.

[31] Moncaz A, Gebresilassie A, Kirstein O, Faiman R, Gebre-Michael T, Hailu A, Warburg A (2013). Attraction of phlebotomine sand flies to baited and non-baited horizontal surfaces. Acta Trop.

